# Supplementary material for: Serum Anti-Spike Antibodies Are Not Affected by Immunosuppressants in SARS-CoV-2 Vaccinations Given to Brazilian Patients with Inflammatory Bowel Disease
Source: Healthcare (Basel). 2023 Oct 19;11(20):2767. doi: 10.3390/healthcare11202767 (PMC10606730; doi:10.3390/healthcare11202767)
Supplement: Supplementary file 1 [file healthcare-11-02767-s001.zip › healthcare-2604732-supplementary.pdf]

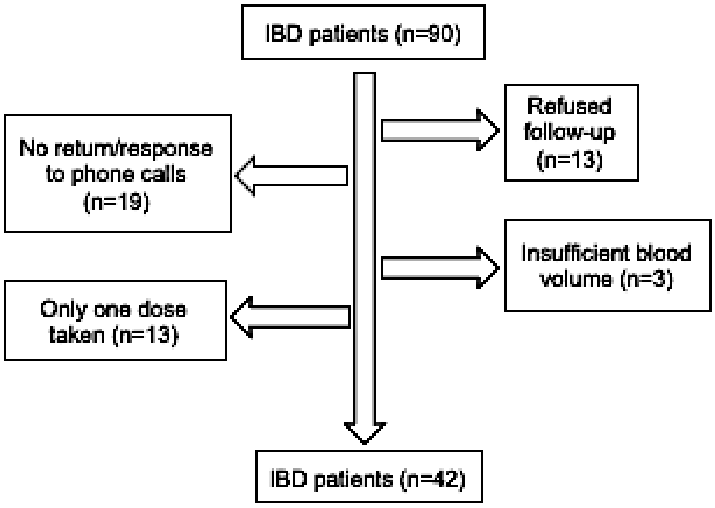

Supplementary Figure S1. Flow chart of patient's recruitment

The diagram shows the path followed by the patients during the selection for the study, with losses and the final number.

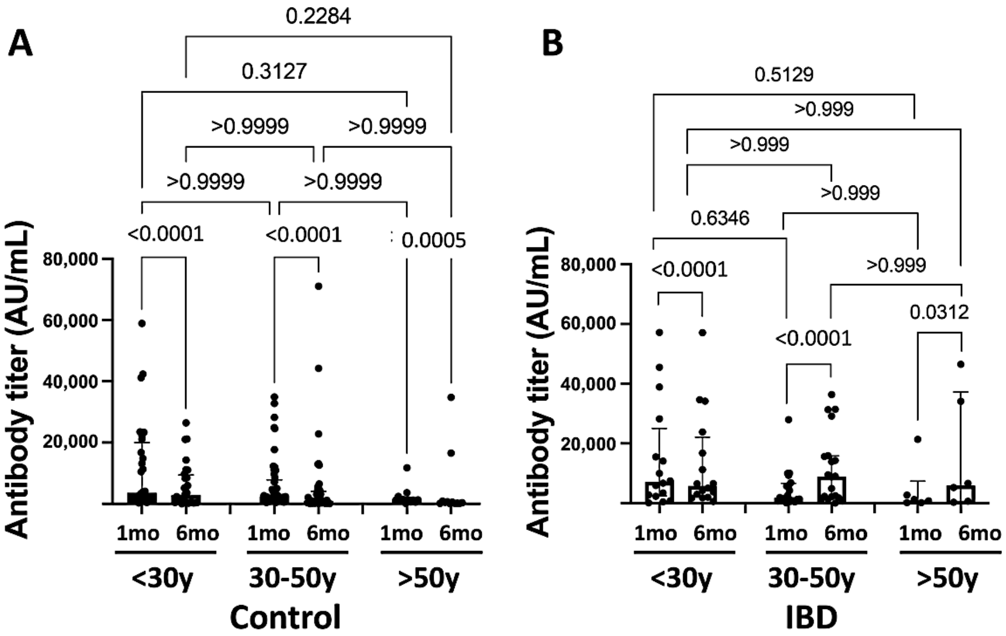

Supplementary Figure S2. SARS-CoV-2 antibody titers in control individuals and patients with IBD, according to different age ranges

The humoral immune response to vaccination against the SARS-CoV-2 spike protein was evaluated by measuring antibody titers 1- and 6-months post-vaccination, in patients with less than 30, between 30 and 50, and more than 50 years. The medians with interquartile ranges and individual values are shown. The analysis was performed using the Kruskal–Wallis test, in which multiple comparisons between the vaccination schemes in each group were performed using Dunn's test and the Wilcoxon matched-pairs signed rank test for pre- and post-vaccination results. IBD, inflammatory bowel disease; SARS-CoV-2, severe acute respiratory syndrome coronavirus 2.
